# Supplementary material for: Low immunogenicity of LNP allows repeated administrations of CRISPR-Cas9 mRNA into skeletal muscle in mice
Source: Nat Commun. 2021 Dec 8;12:7101. doi: 10.1038/s41467-021-26714-w (PMC8654819; doi:10.1038/s41467-021-26714-w)
Supplement: Supplementary file 1 — Supplementary Information [file 41467_2021_26714_MOESM1_ESM.pdf]

Supplementary Fig. 1

a

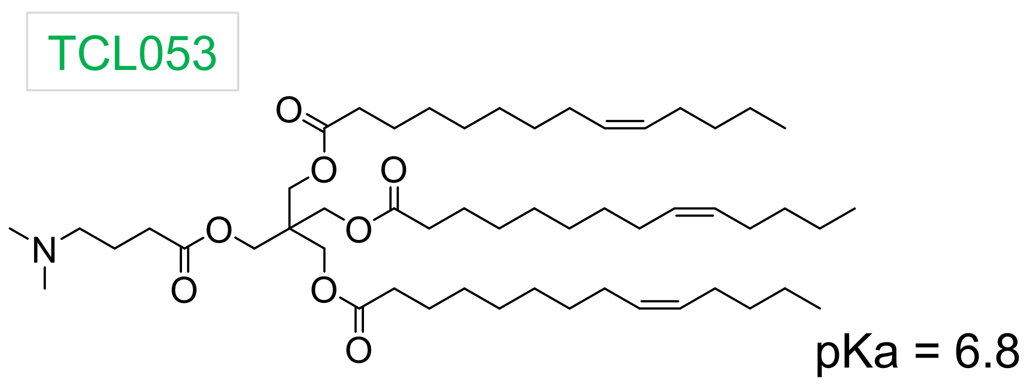

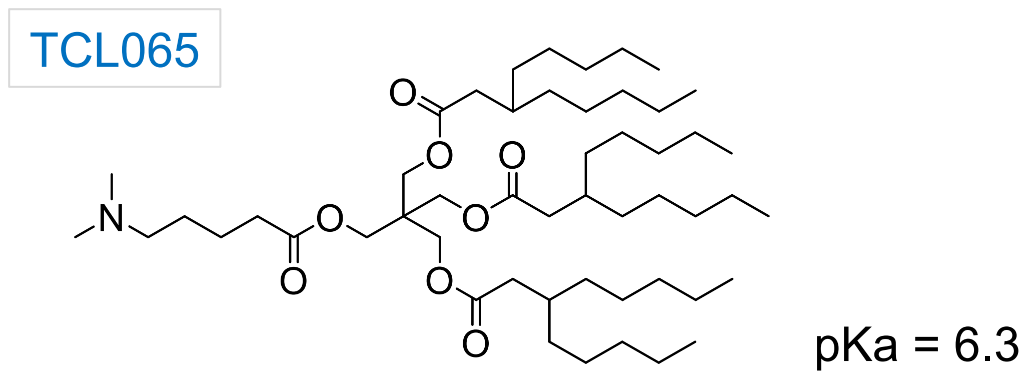

b

LNP	Composition	Encapsulation (%)	Size (nm)	PDI
TCL053-LNP-I	TCL053 : DPPC : Cholesterol : DMG-PEG = 60 : 10.6 : 28.7 : 0.7	95.8	88.6	0.040
TCL053-LNP-II	TCL053 : DPPC : Cholesterol : DMG-PEG = 60 : 10.6 : 28 :1.4	98.5	73.1	0.061
TCL053-LNP-III	TCL053 : DOPE : Cholesterol : DMG-PEG = 60 : 10.6 : 28.7 : 0.7	97.9	95.1	0.026
TCL053-LNP-IV	TCL053 : DOPE : Cholesterol : DMG-PEG = 60 : 10.6 : 28 : 1.4	96.1	78.9	0.025
TCL065-LNP-I	TCL065 : DPPC : Cholesterol : DMG-PEG = 60 : 10.6 : 28.7 : 0.7	96.7	73.7	0.051
TCL065-LNP-II	TCL065 : DPPC : Cholesterol : DMG-PEG = 60 : 10.6 : 28 : 1.4	97.5	71.8	0.044
TCL065-LNP-III	TCL065 : DOPE : Cholesterol : DMG-PEG = 60 : 10.6 : 28.7 : 0.7	97.8	90.8	0.101
TCL065-LNP-IV	TCL065 : DOPE : Cholesterol : DMG-PEG = 60 : 10.6 :28 : 1.4	97.2	79.0	0.042

c

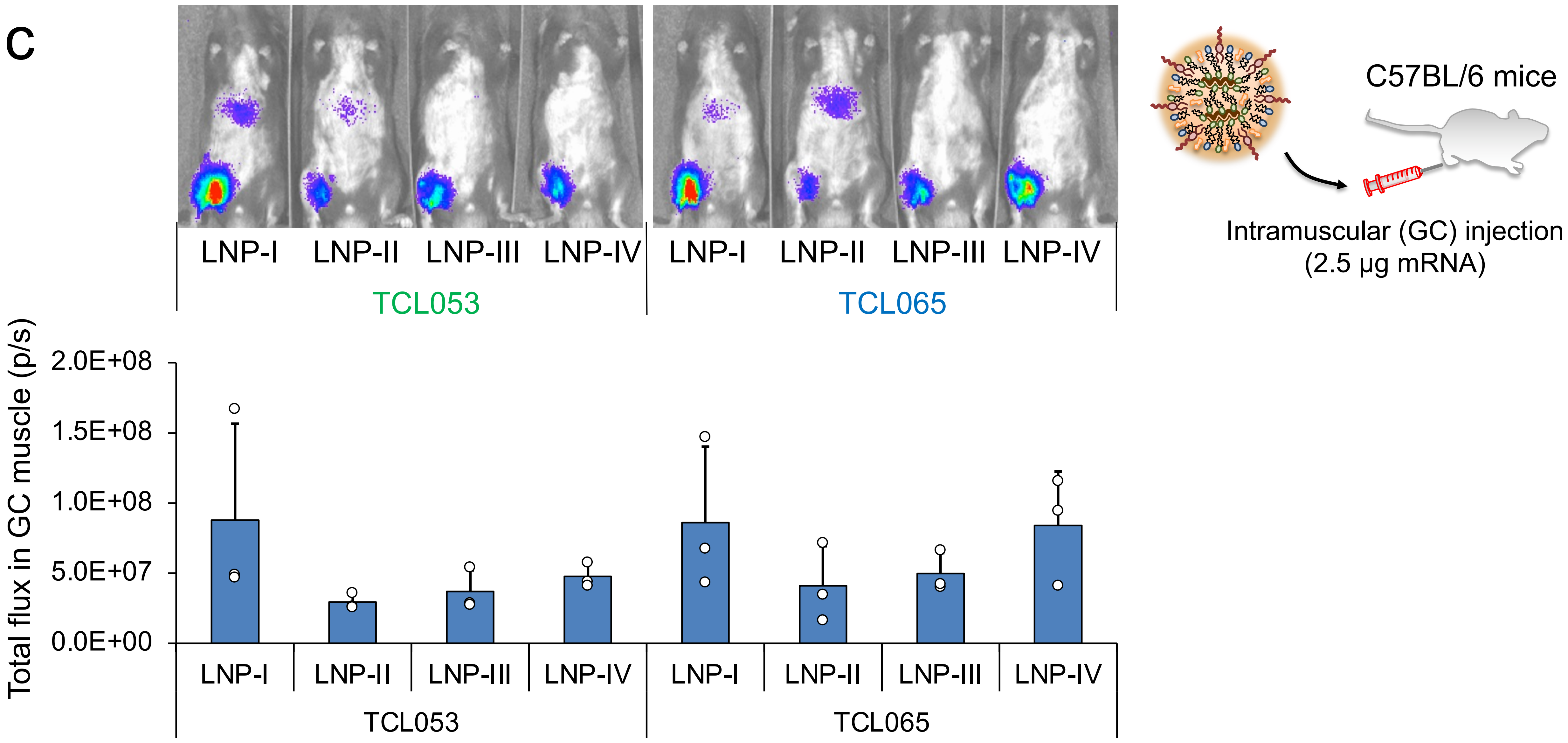

Supplementary Fig. 1 | Formulation and characterization of LNPs for Luc mRNA delivery.
(a) Chemical structures and pKa values of the ionizable lipids TCL053 and TCL065.
(b) Luciferase mRNA was encapsulated into various formulations of LNP. Dynamic light scattering was used to assess the size, and polydispersity index (PDI) of the LNPs. Encapsulation efficiency of mRNA was measured by Ribogreen assay.
(c) LNP-Luc mRNA was injected into the GC muscle in C57BL/6J mice. Four hours later, luciferin was injected intraperitoneally to measure the luciferase activity using IVIS. Quantification of the luciferase signal (total flux, photons per second) at the GC muscle from 3 mice is shown in a bar graph. Mean ± S.D.

Supplementary Fig. 2

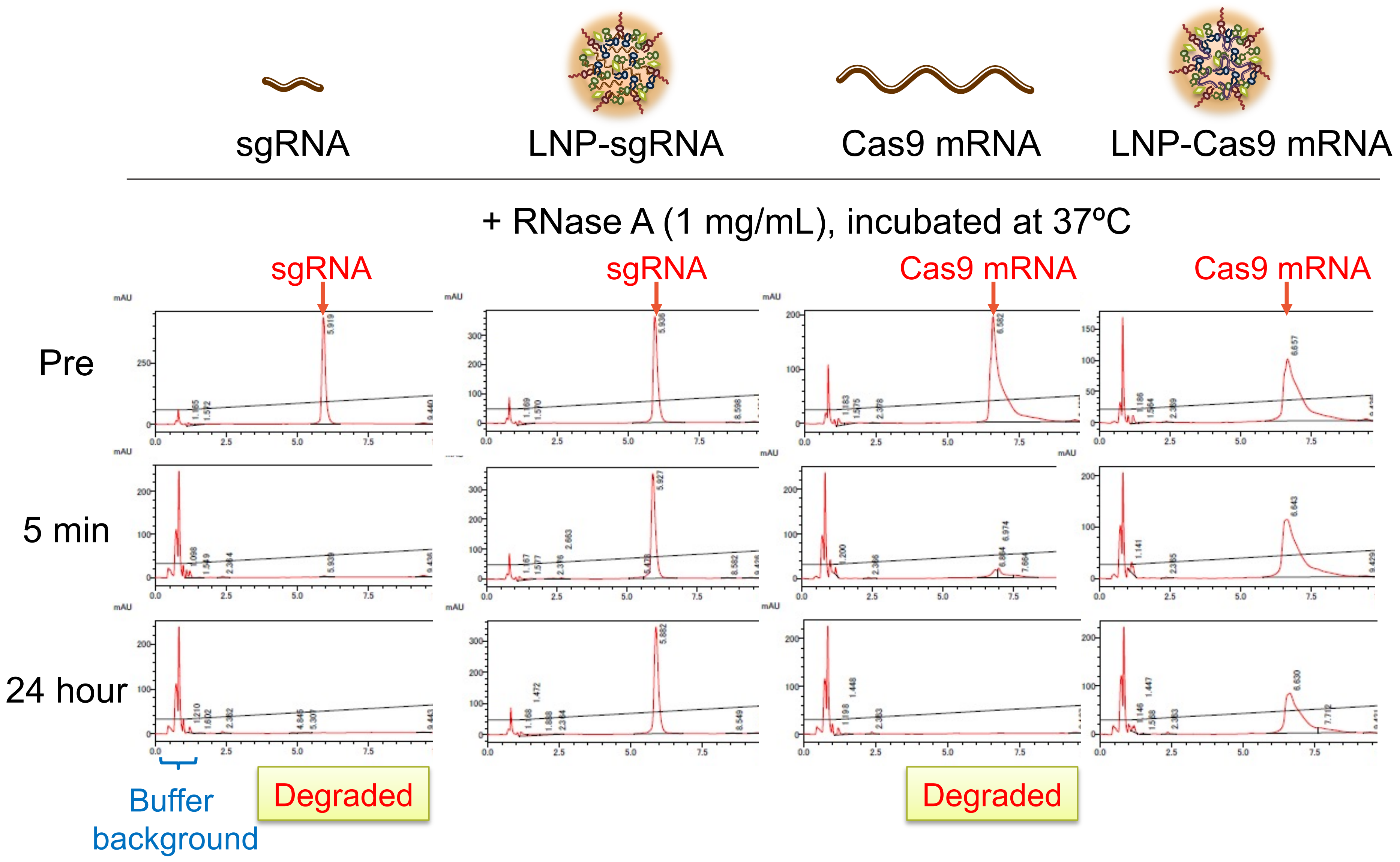

Supplementary Fig. 2 | Stability of LNP-encapsulated RNA after RNase A challenge. Mouse Rosa26 targeting sgRNA, Cas9 mRNA and an LNP formulation were incubated with 1 mg/mL RNase A (RNA endonuclease) at 37°C. After 5 minutes or 24 hours of incubation, sgRNA or Cas9 mRNA were extracted and analyzed by reverse phase HPLC. Naked sgRNA and mRNA quickly degraded within 5 min after the RNase A challenge, but sgRNA and mRNA encapsulated in LNP remained intact for 24 hours.

Supplementary Fig. 3

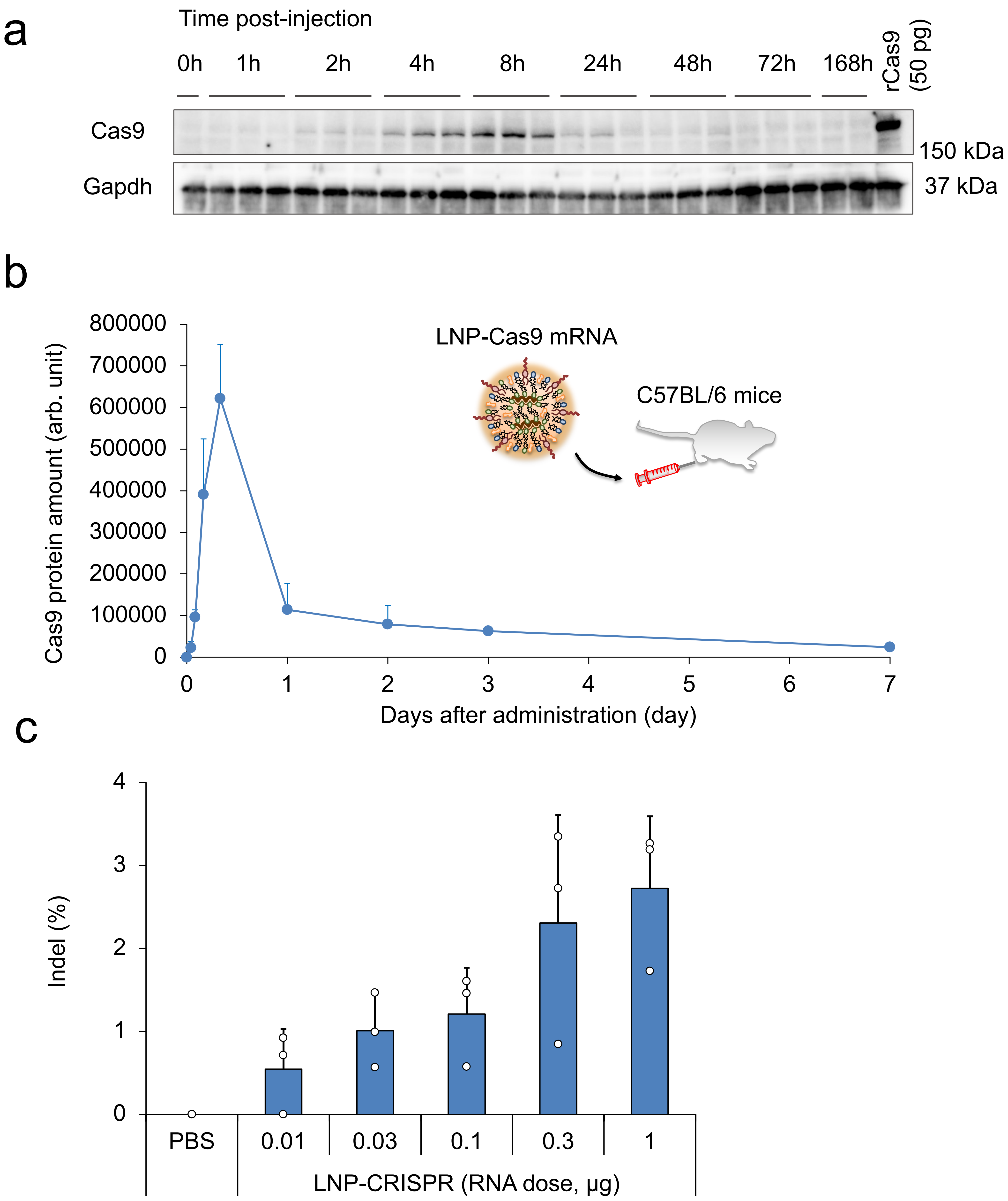

Supplementary Fig. 3 | Kinetics of Cas9 expression after the intramuscular injection of LNP-Cas9 mRNA.

(a) Western blot analysis of Cas9 and Gapdh protein in GC of mice treated with LNP-Cas9 mRNA (2.5 μg) at 0 (no-injection control), 1, 2, 4, 8, 24, 48, 72, or 168 hours post-injection. n = 3 mice, except for 0 (n = 1) and 168 hour (n = 2) samples. rCas9 is recombinant Cas9 protein spike in control (n= 1).

(b) Quantified Cas9 protein amount from (a). Data shown as mean + S.D. with an arbitrary unit (arb. unit). n = 3 mice, except for 0 (n = 1) and 168 hour (n = 2) samples.

(c) Genome editing efficacy of LNP-CRISPR, a mixture of LNP-sgRNA targeting mouse Rosa26 and LNP-Cas9 mRNA. LNP-CRISPR (0.01 - 1 μg of each RNA component) was injected into the GC muscle of C57BL/6J mice. The editing efficiency of genomic DNA was measured by T7E1 assay. Data are shown as mean + S.D. (n = 3 mice).

Supplementary Fig. 4

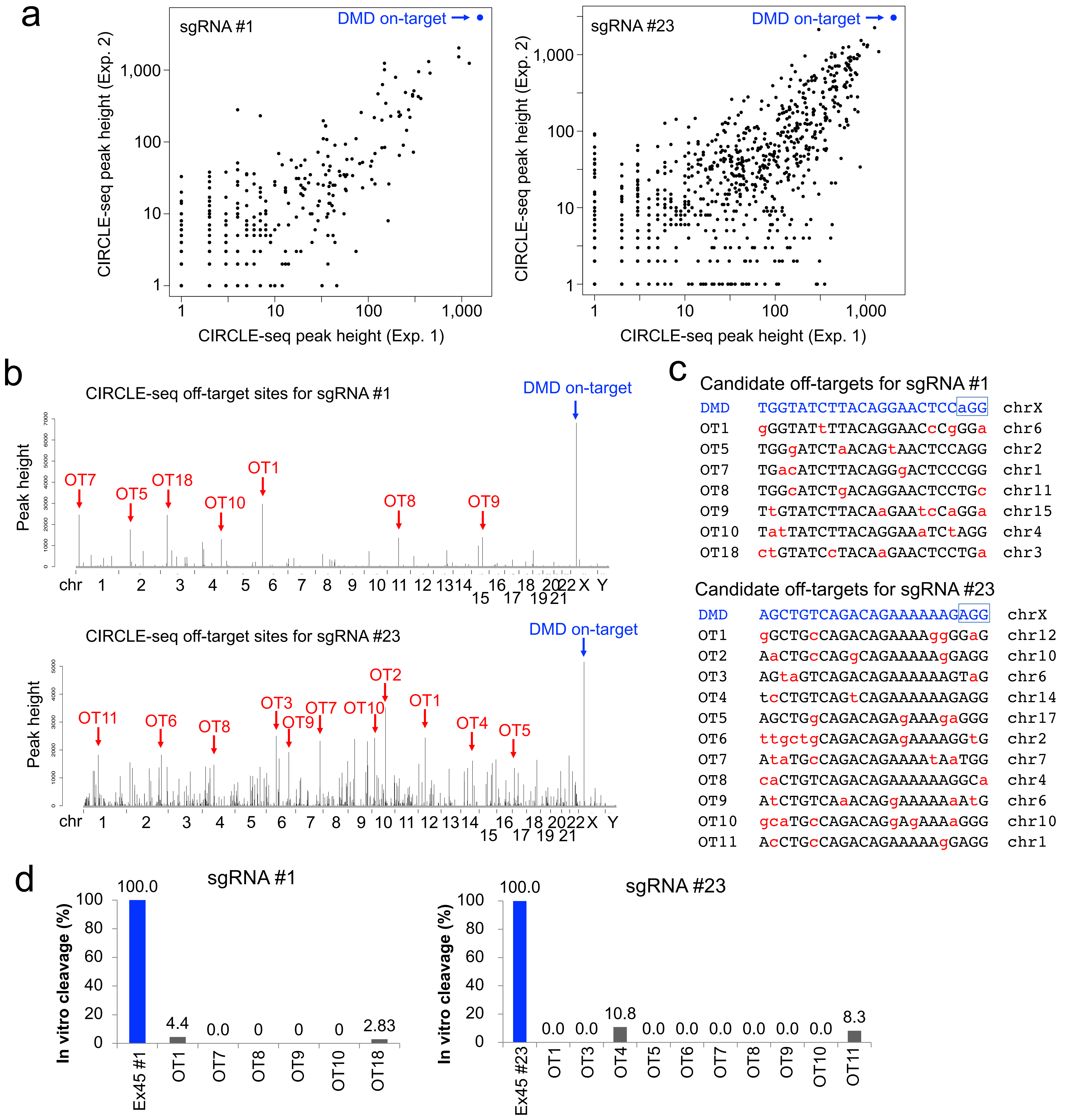

Supplementary Fig. 4 | Cell-free CIRCLE-seq assay to search for candidate off-target sites.

(a) Two independent CIRCLE-seq experiments show reproducible peak identification, including the DMD on-target with the highest peak. (b) Identified CIRCLE-seq peaks show putative on- and off-target sites over chromosomes. Red arrows indicate positions of off-target (OT) sites which were analyzed in the following T7EI assay. (c) The identified putative sgRNA binding sites within each on- or off-target peak by Cas-OFFinder analysis. (d) The *in vitro* cleavage assay using amplified on- or off-target DNA fragments.

Supplementary Fig. 5

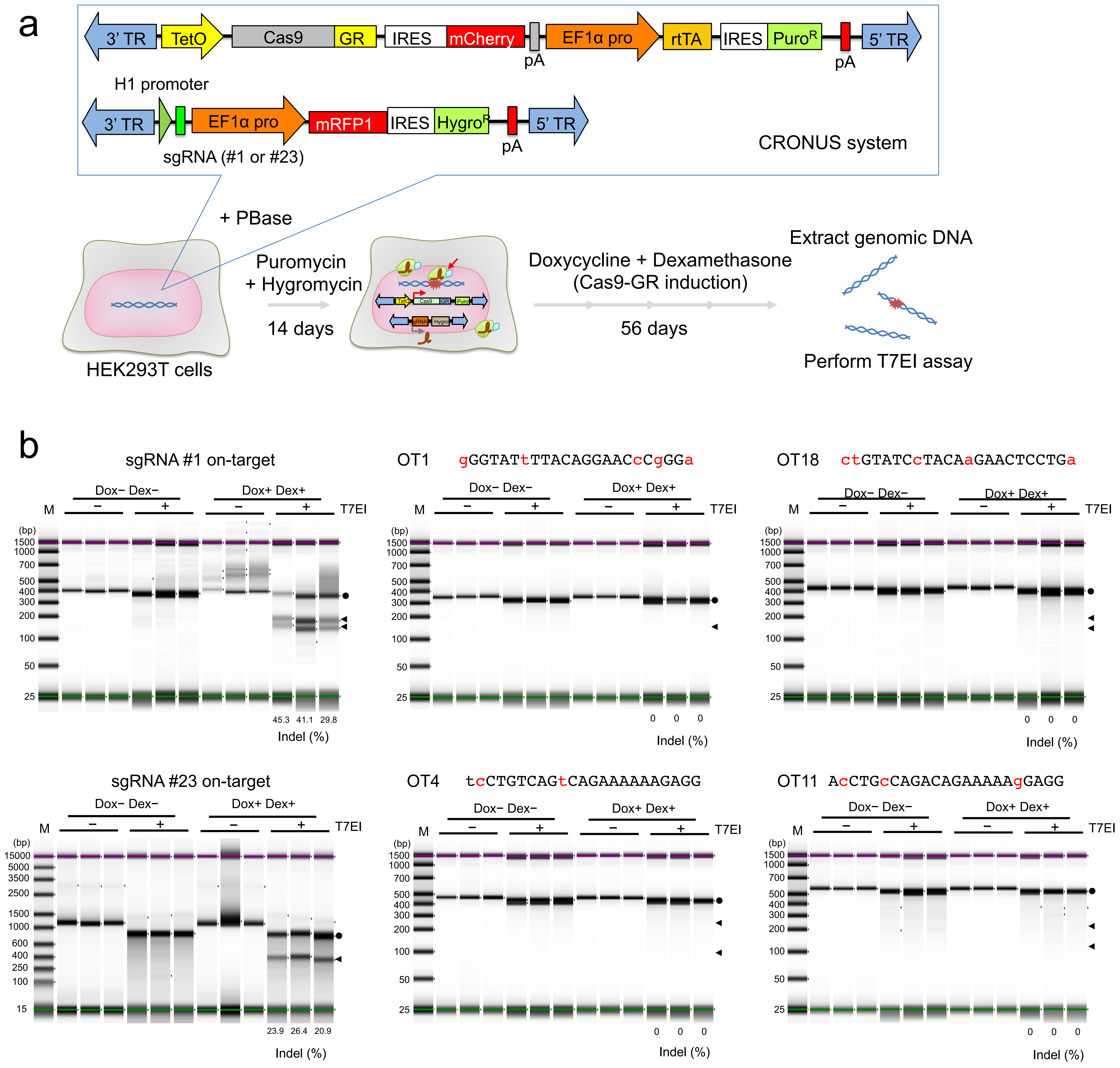

Supplementary Fig. 5 | Prolonged Cas9/gRNA treatment to assess off-target mutagenesis in human cells

(a) Schematic of the experimental design. Cas9-GR and sgRNA expression cassettes were stable integrated by the CRONUS *piggyBac* vector in human HEK293T cells and the expression of Cas9-GR was induced by doxycycline and dexamethasone for 56 days.

(b) T7EI assay to detect genomic indels using CRONUS-293T cells treated with Cas9/gRNA for 56 days. On-target for sgRNA #1 and #23 showed clear indels when T7EI was added, whereas no cleavage detected for off-target sites that were identified by the *in vitro* cleavage assay in Sup. Fig. 4d.

Supplementary Fig. 6

a

LNP	Composition	Encapsulation (%)	Size (nm)	PDI
TCL053-LNP-I	TCL053 : DPPC : Cholesterol : DMG-PEG = 60 : 10.6 : 28.7 : 0.7	91.6	108.6	0.132
TCL053-LNP-V	TCL053 : DPPC : Cholesterol : DMG-PEG = 60 : 10.6 : 27.3 : 2.1	96.0	79.1	0.128
MC3-LNP-I	MC3-DLin-DMA : DPPC : Cholesterol : DMG-PEG = 60 : 10.6 : 28.7 : 0.7	95.8	88.6	0.147
MC3-LNP-V	MC3-DLin-DMA : DPPC : Cholesterol : DMG-PEG = 60 : 10.6 : 27.3 : 2.1	96.2	72.1	0.167
MC3-LNP-VI	MC3-DLin-DMA : DSPC : Cholesterol : DMG-PEG = 50 : 10 : 38.5 : 1.5	96.3	98.4	0.100

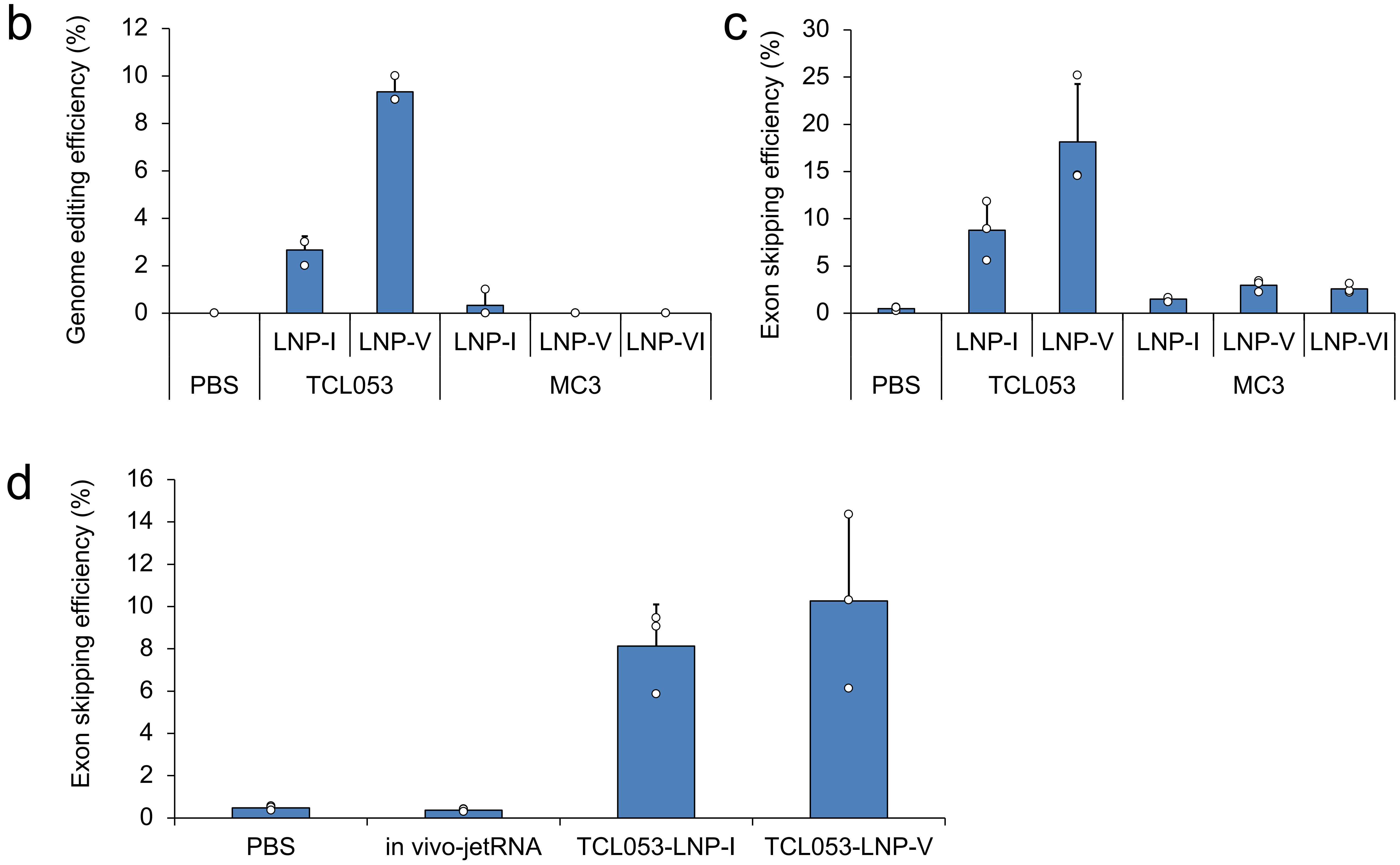

Supplementary Fig. 6 | Comparing the efficacy of TCL053-LNP with benchmark gene delivery systems

(a) Cas9 mRNA, hEx45 sgRNA #1 and sgRNA #23 were encapsulated into TCL053-LNP or MC3-LNP with several lipid compositions. Dynamic light scattering was used to assess the size and polydispersity index (PDI) of the LNPs. Encapsulation efficiency of mRNA was measured by Ribogreen assay.

(b, c) Genome editing efficiency was measured by droplet digital PCR (b) and exon skipping efficiency was measured by qRT-PCR (c) a week after injection of TCL053-LNP-CRISPR or MC3-LNP-CRISPR (20 µg total RNA) into TA of hEx45KI-mdx44 mice. Data are shown as mean + S.D. (n = 3 mice).

(d) Exon skipping efficiency was measured by qRT-PCR a week after injection of TCL053-LNP-CRISPR or in vivo-jet RNA-CRISPR (Polyplus, Illkirch, France) (5 µg of total RNA) into TA of hEx45KI-mdx44 mice. Data are shown as mean + S.D. (n = 3 mice).

Supplementary Fig. 7

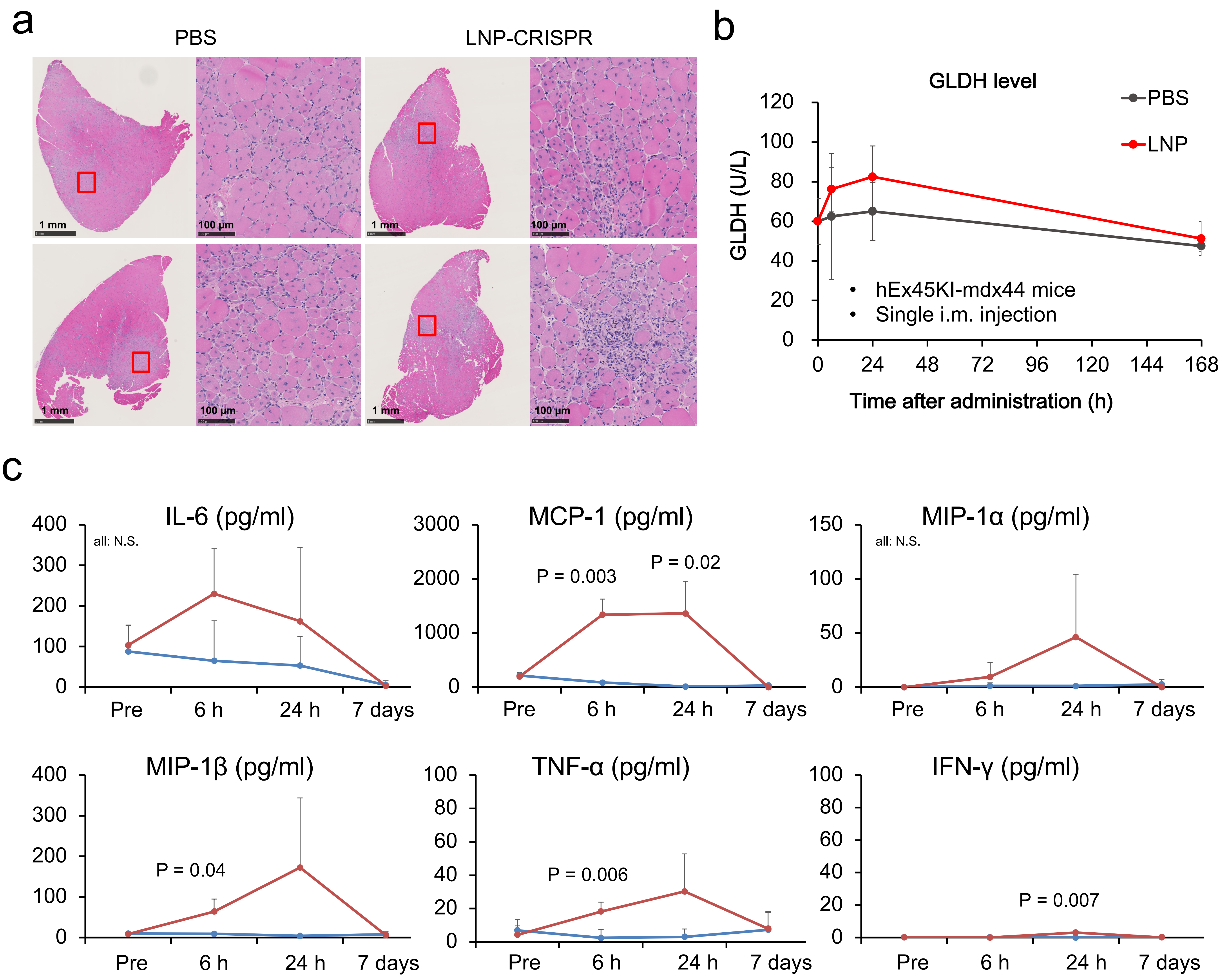

Supplementary Fig. 7 | Safety assessment of LNP-CRISPR

(a) LNP-CRISPR (5 µg of each sgRNA and 10 µg of Cas9 mRNA) was intramuscularly administered to TA muscle in hEx45KI-mdx44 mice (DMD model mice). Seven days after the single administration, muscle tissue was extracted, and historiological assessment was performed after H&E staining. Inflammation and fibrosis was observed in both PBS and LNP-CRISPR injected tissues, and most significant areas (red rectangle) are shown in magnified view on the right. Representative image in each group of 4 mice is shown.

(b) After intramuscular injection of LNP-CRISPR, blood samples were collected at 0 (pre-treatment), 4, 24, and 168 hours (7 days) to measure GLDH (glutamate dehydrogenase) levels in plasma, as a liver injury marker. n = 4 mice, mean ± S.D. There was no statistically significant difference between PBS and LNP, or 0 hour and other sampling points by Student's or Aspin-Welch's t-test. U/L: unit per little.

(c) Same as the above LNP injection, blood samples were collected at 0 (pre-treatment), 4, 24, and 168 hours (7 days) to measure various cytokine levels (MCP-1, MIP-1α/β, IL-6, TNF-α, and IFN-γ) in plasma, by using a Cytometric Bead array (BD Biosciences). n = 4 mice, Mean ± S.D. For significant results, data were analyzed by the Aspin-Welch t test. Differences were considered statistically significant when *p* values were ≤ 0.05.

Supplementary Fig. 8

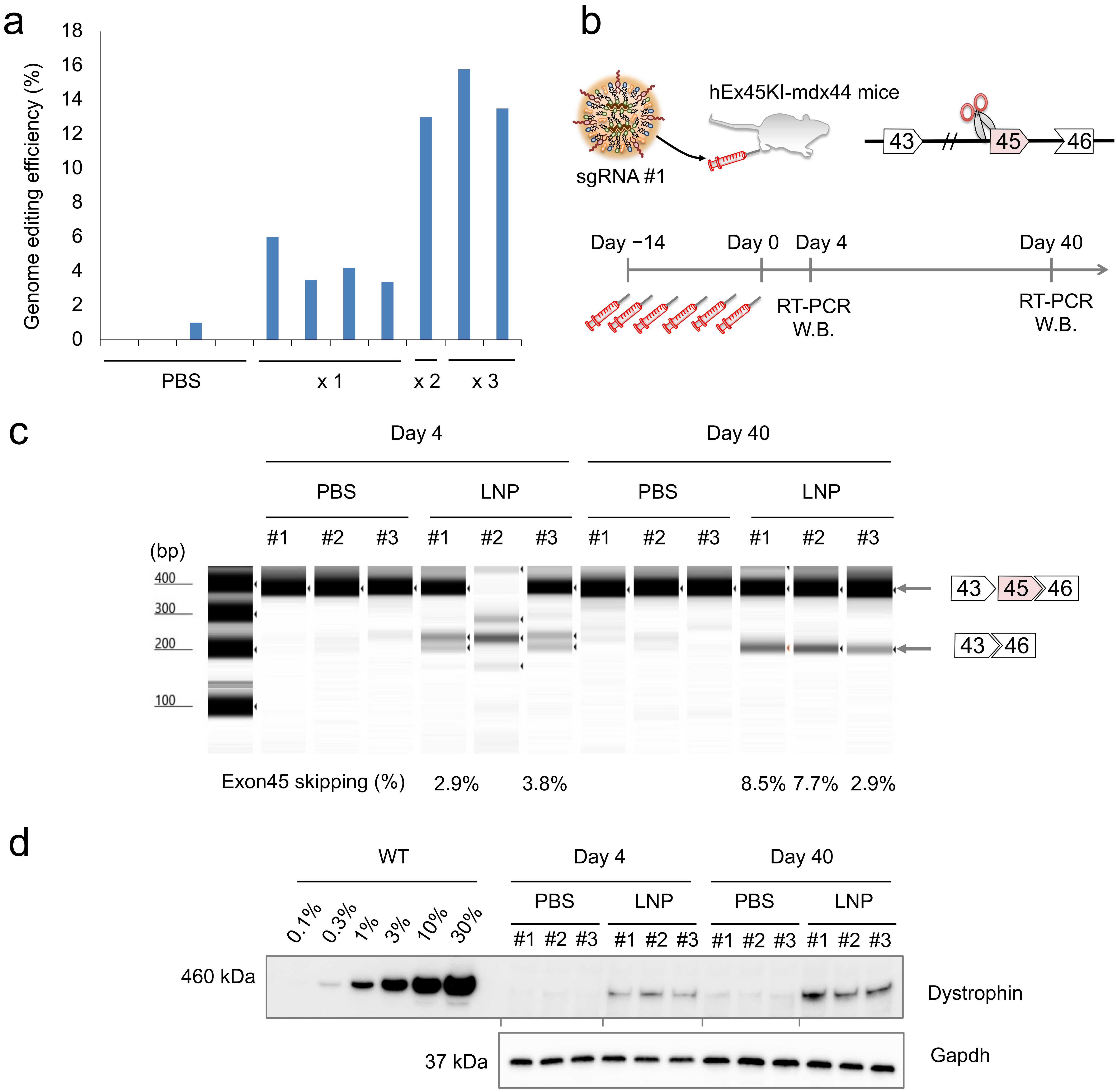

Supplementary Fig. 8 | Repeated administrations of LNP-CRISPR with single sgRNA.
(a) Genome editing efficacy in GC of hEx45 KI mice was measured by droplet digital PCR after one, two or three times injections (every other day) of LNP-Cas9 mRNA (10 μg) and LNP-hEx45 sgRNA#1 (10 μg). n = 4 for PBS and one-time injection groups, n = 1 for two-times injection group, and n = 2 for three-times injection group.
(b) LNP-Cas9 mRNA (3 μg) and LNP- hEx45 sgRNA #1 (3 μg) were repeatedly injected into GC of hEx45KI-mdx44 mice 6 times over 2 weeks.
(c) Exon skipping efficiency in GC was measured by RT-PCR 4 and 40 days after the final injection. LNP-injected mouse #2 failed with the RT-PCR reaction, presumably due to the low quality of mRNA. n = 3 mice.
(d) The expression of dystrophin protein by western blot analysis in GC 4 and 40 days after the final injection. n = 3 mice.

Supplementary Fig. 9

a

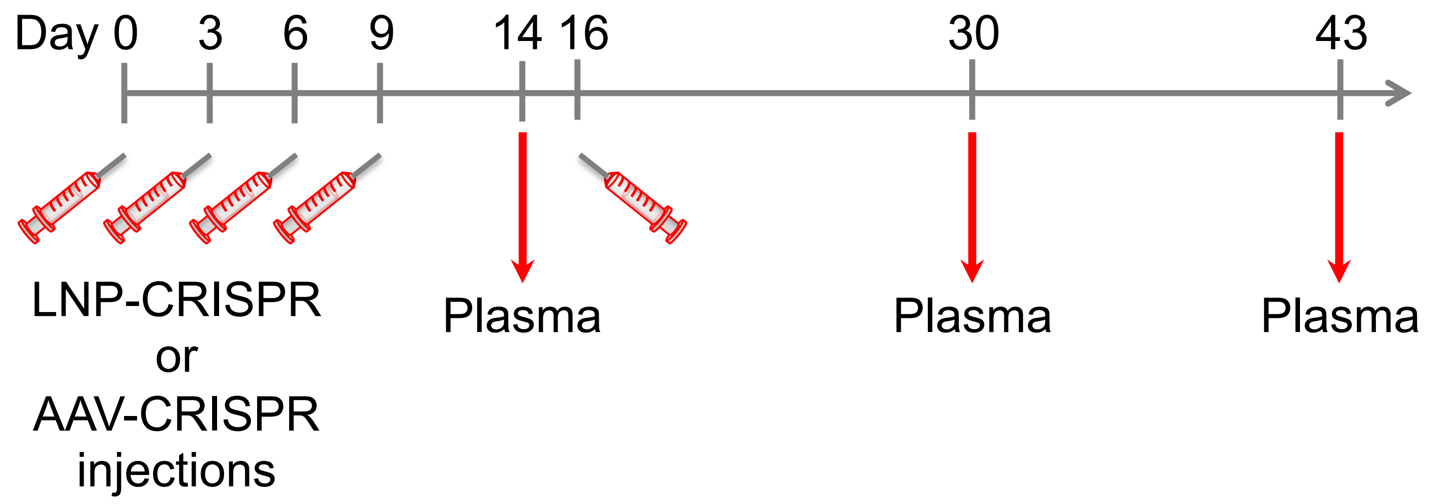

b

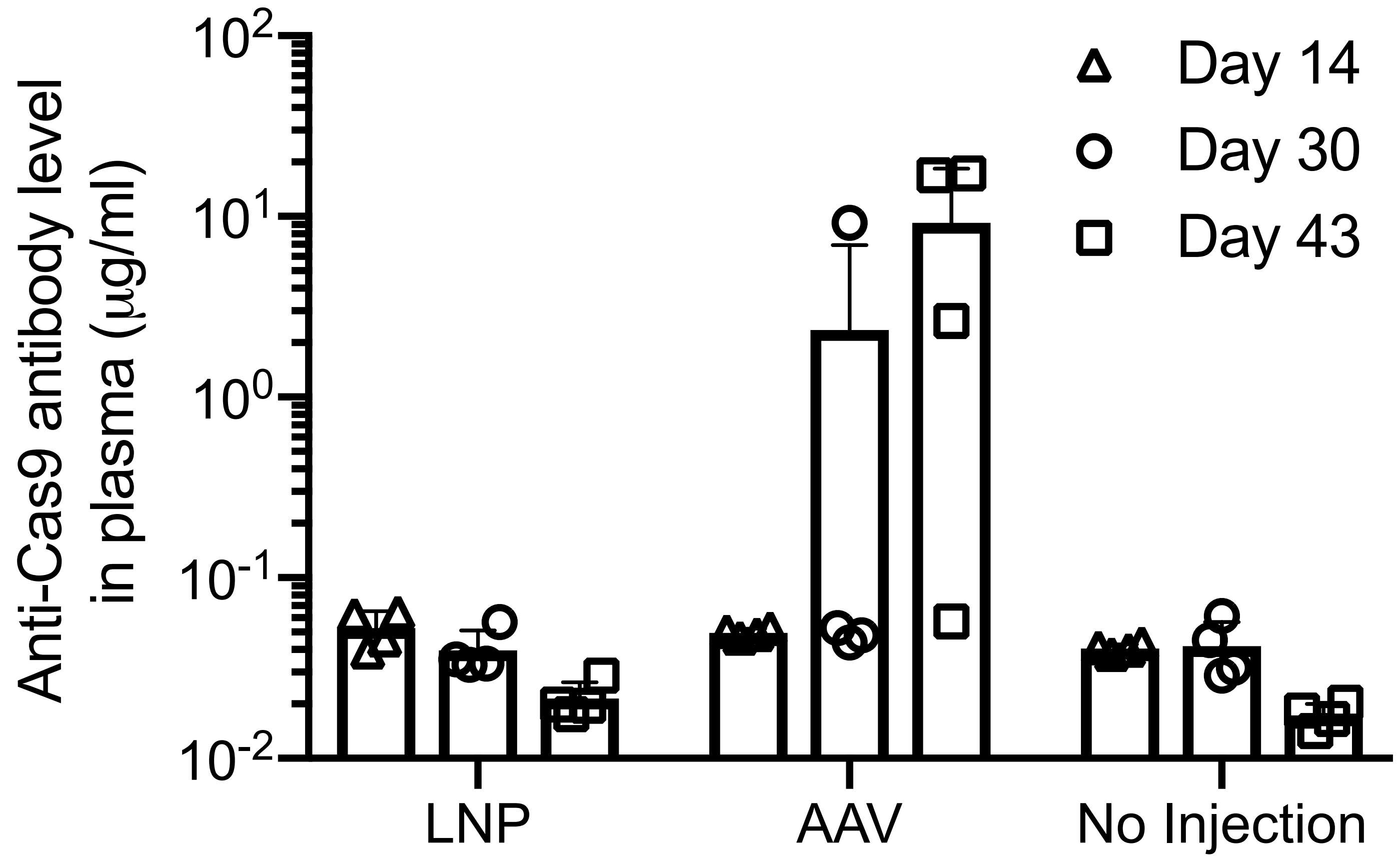

Supplementary Fig. 9 | Anti-SpCas9 antibodies in plasma induced by LNP and AAV injection.

(a) We performed multiple injections (total 5 times) of LNP-CRISPR (6 μg mRNA + sgRNA) or AAV-CRISPR (2×10^{11} v.g.) into GC muscle of the Luc-reporter mice, and corrected plasma samples at the indicated time points.

(b) We measured the level of anti-SpCas9 antibody in mouse plasma by ELISA. Mean ± S.D. n = 4 mice.

Supplementary Fig. 10

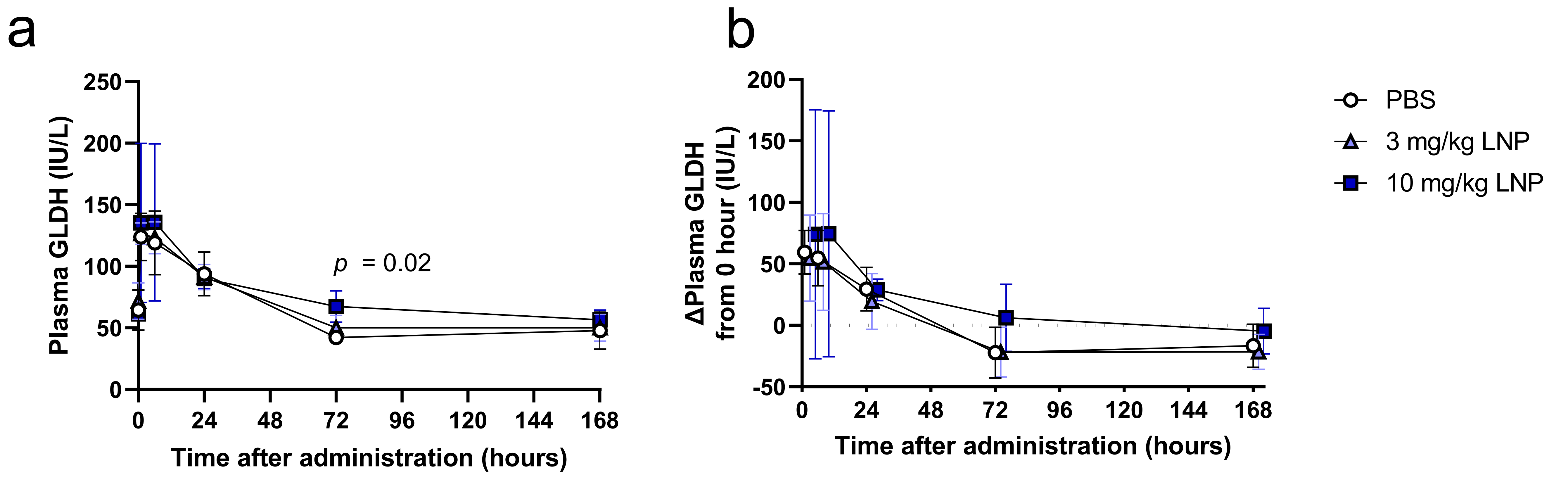

Supplementary Fig. 10 | Plasma GLDH induced by Limb perfusion

(a) Plasma GLDH level was measured at pre (0 hour), 1, 6, 24, 72 and 168 hours after administration of PBS or LNP-CRISPR by limb perfusion in hEx45KI-mdx44 mice. Data are represented as mean ± S.D. (n = 4 mice). *p* value by Bonferroni adjusted two-sided Williams' test showed significantly different from PBS injection group.

(b) The same experimental result from (a) is plotted as ΔPlasma GLDH to show the change from pre-administration. Data are presented as mean ± 95% confidence interval. The plots are intentionally staggered for X-axis for ease of viewing.
