# Supplementary material for: Low immunogenicity of LNP allows repeated administrations of CRISPR-Cas9 mRNA into skeletal muscle in mice
Source: Nat Commun. 2021 Dec 8;12:7101. doi: 10.1038/s41467-021-26714-w (PMC8654819; doi:10.1038/s41467-021-26714-w)
Supplement: Supplementary file 2 — Reporting Summary [file 41467_2021_26714_MOESM2_ESM.pdf]

nature research

Corresponding author(s): Akitsu Hotta

Last updated by author(s): Sep 21, 2021

Reporting Summary

Nature Research wishes to improve the reproducibility of the work that we publish. This form provides structure for consistency and transparency in reporting. For further information on Nature Research policies, see our Editorial Policies and the Editorial Policy Checklist.

Statistics

For all statistical analyses, confirm that the following items are present in the figure legend, table legend, main text, or Methods section.

n/a	Confirmed	
☐	☒	The exact sample size (n) for each experimental group/condition, given as a discrete number and unit of measurement
☐	☒	A statement on whether measurements were taken from distinct samples or whether the same sample was measured repeatedly
☐	☒	The statistical test(s) used AND whether they are one- or two-sided<br>*Only common tests should be described solely by name; describe more complex techniques in the Methods section.*
☒	☐	A description of all covariates tested
☒	☐	A description of any assumptions or corrections, such as tests of normality and adjustment for multiple comparisons
☐	☒	A full description of the statistical parameters including central tendency (e.g. means) or other basic estimates (e.g. regression coefficient) AND variation (e.g. standard deviation) or associated estimates of uncertainty (e.g. confidence intervals)
☐	☒	For null hypothesis testing, the test statistic (e.g. F, t, r) with confidence intervals, effect sizes, degrees of freedom and P value noted<br>*Give P values as exact values whenever suitable.*
☒	☐	For Bayesian analysis, information on the choice of priors and Markov chain Monte Carlo settings
☒	☐	For hierarchical and complex designs, identification of the appropriate level for tests and full reporting of outcomes
☒	☐	Estimates of effect sizes (e.g. Cohen's d, Pearson's r), indicating how they were calculated

Our web collection on statistics for biologists contains articles on many of the points above.

Software and code

Policy information about availability of computer code

Data collection	CIRCLE-seq data was collected by illumina NextSeq sequencer.
Data analysis	For CIRCLE-seq analysis, following publicly available software was used: cutadapt software (Version 2.4 with Python 3.4.10), BWA-MEM (version: bwa-0.7.17-r1188), samtools (Version 0.1.19-44428cd), bedtools (Version 2.27.1), and R (Version 3.6.1).<br><br>Statistical analysis was conducted by EXSUS 2014 (Version 8.0, SAS 9.3 TS Levle1M2) from SAS Institute, Inc, NC.

For manuscripts utilizing custom algorithms or software that are central to the research but not yet described in published literature, software must be made available to editors and reviewers. We strongly encourage code deposition in a community repository (e.g. GitHub). See the Nature Research guidelines for submitting code & software for further information.

Data

Policy information about availability of data

All manuscripts must include a data availability statement. This statement should provide the following information, where applicable:

- Accession codes, unique identifiers, or web links for publicly available datasets
- A list of figures that have associated raw data
- A description of any restrictions on data availability

All sequence data used in CIRCLE-seq will be available from NCBI SRA database (accession number: PRJNA615771]).

April 2020

Field-specific reporting

Please select the one below that is the best fit for your research. If you are not sure, read the appropriate sections before making your selection.

- [x] Life sciences
- [ ] Behavioural & social sciences
- [ ] Ecological, evolutionary & environmental sciences

For a reference copy of the document with all sections, see nature.com/documents/nr-reporting-summary-flat.pdf

Life sciences study design

All studies must disclose on these points even when the disclosure is negative.

Sample size	All in vitro and in cell culture experiments were performed in technical triplicates. For in vivo experiments, n= 3 - 5 mice were used for analysis. No sample size calculation was performed. Sample sizes were estimated based on standards of the field.
Data exclusions	For IVIS imaging analysis, dead mice were excluded. For CIRCLE-seq analysis, unmapped reads and non-specific peaks (no defined cutting edge) were excluded.
Replication	Multiple mice or cell samples were examined for each experiment. All findings can be reproduced with the same methodology in the multiple experiments. Key experiments (i.e. LNP injection and assessment of in vivo genome editing) were confirmed by more than two researchers.
Randomization	Littermate mice were classified based on body weight and randomly separated into experimental groups, so that each group has similar average body weight. No randomization was done for cell based experiments because multiple conditions are well controlled among multiple samples.
Blinding	Blinding was not used in this study, to avoid sample misidentification. Bias cannot alter the outcome of the delivery efficiencies and genome editing patterns.

Reporting for specific materials, systems and methods

We require information from authors about some types of materials, experimental systems and methods used in many studies. Here, indicate whether each material, system or method listed is relevant to your study. If you are not sure if a list item applies to your research, read the appropriate section before selecting a response.

Materials & experimental systems

n/a	Involved in the study	
[ ]	[x]	Antibodies
[ ]	[x]	Eukaryotic cell lines
[x]	[ ]	Palaeontology and archaeology
[ ]	[x]	Animals and other organisms
[x]	[ ]	Human research participants
[x]	[ ]	Clinical data
[x]	[ ]	Dual use research of concern

Methods

n/a	Involved in the study	
[x]	[ ]	ChIP-seq
[x]	[ ]	Flow cytometry
[x]	[ ]	MRI-based neuroimaging

Antibodies

Antibodies used

<Western blot>
Rabbit polyclonal anti-dystrophin (cat #ab15277, Abcam, Cambridge, UK; 2,000-fold dilution),
Rabbit anti-GAPDH (cat #2118, Cell Signaling Technology, Danvers, MA; 4,000-fold dilution),
Rabbit polyclonal anti-Cas9 (cat #632607, Takara Bio; 1,000-fold dilution),
anti-rabbit IgG HRP-linked whole antibody (cat#NA934-aML, GE Healthcare, Chicago, IL, 5,000-fold dilution for dystrophin, 1,000- or 5,000-fold dilution for GAPDH or 1,000-fold dilution for Cas9)

<Immunohistochemistry>
polyclonal anti-dystrophin (cat #ab15277, Abcam, Cambridge, UK; 100-fold dilution)
polyclonal anti-Laminin 2 alpha (cat #ab11576, Abcam, Cambridge, UK; 1,000-fold dilution)
rabbit anti-rat IgG (H + L), biotinylated (cat#BA-4000, Vector laboratories, CA, 200-fold dilution)
goat anti-Rabbit IgG (H;L) Highly Cross-Adsorbed Secondary Antibody, Alexa Fluor 568 (cat#A11036, Thermo Ficher Scientific, Waltham, MA, 200-fold dilution)

Validation

All the antibodies used in the study were validated by the manufacture.
anti-dystrophin: https://www.abcam.co.jp/dystrophin-antibody-ab15277.html
anti-GAPDH: https://www.cellsignal.jp/products/primary-antibodies/gapdh-14c10-rabbit-mab/2118
anti-Cas9: https://www.takarabio.com/products/gene-function/gene-editing/crispr-cas9/cas9-antibodies

anti-rabbit IgG HRP-linked whole antibody : https://www.gelifesciences.com/en/us/shop/protein-analysis/blotting-and-detection/blotting-standards-and-reagents/amersham-ecl-hrp-conjugated-antibodies-p-06260
anti-Laminin 2 alpha: https://www.abcam.co.jp/laminin-2-alpha-antibody-4h8-2-ab11576.html
anti-rat IgG: https://vectorlabs.com/biotinylated-rabbit-anti-rat-igg-antibody.html
anti-rabbit IgG: https://www.thermofisher.com/antibody/product/Goat-anti-Rabbit-IgG-H-L-Highly-Cross-Adsorbed-Secondary-Antibody-Polyclonal/A-11036

Eukaryotic cell lines

Policy information about cell lines

Cell line source(s)	HEK293 cells were purchased from ATCC (CRL-3216). DMD-iPS cells (clone CiRA00111) is available from RIKEN BRC (HPS0383).
Authentication	Pathogenic deletion of exon 44 in DMD-iPS cells (clone CiRA00111) is validated by PCR genotyping. None of the other lines were authenticated.
Mycoplasma contamination	Cells are confirmed to be mycoplasma negative using MycoAlert Mycoplasma Detection Kit (Lanza).
Commonly misidentified lines (See ICLAC register)	None of the cell lines used in the study is listed as Commonly misidentified lines by ICLAC.

Animals and other organisms

Policy information about studies involving animals; ARRIVE guidelines recommended for reporting animal research

Laboratory animals	Species: Mus muscles (mouse), Strain: C57BL/6J, sex: Male, age: 7 to 13 weeks of age. Mice were housed under SPF condition with free-food and water supply with 12 hour dark/light cycle, controlled temperature (around 23ºC) and controlled humidity (around 55%).
Wild animals	The study did not involve wild animals.
Field-collected samples	The study did not involve field-collected samples.
Ethics oversight	All mouse experiments were evaluated and approved by the Institutional Animal Care and Use Committee in Takeda Pharmaceutical Company Limited.

Note that full information on the approval of the study protocol must also be provided in the manuscript.
